# Anti-Melanogenic Activity of Gagunin D, a Highly Oxygenated Diterpenoid from the Marine Sponge *Phorbas* sp., via Modulating Tyrosinase Expression and Degradation

**DOI:** 10.3390/md14110212

**Published:** 2016-11-17

**Authors:** Ho Yeon Lee, Eun Jeong Jang, Song Yi Bae, Ju-eun Jeon, Hyen Joo Park, Jongheon Shin, Sang Kook Lee

**Affiliations:** College of Pharmacy, Natural Products Research Institute, Seoul National University, Seoul 151-742, Korea; smilehy35@snu.ac.kr (H.Y.L.); jangjenny@snu.ac.kr (E.J.J.); sybae@mit.edu (S.Y.B.); jueun@stanford.edu (J.J.); phj00@snu.ac.kr (H.J.P.); shinj@snu.ac.kr (J.S.)

**Keywords:** Gagunin D, melanogenesis, tyrosinase, melan-a

## Abstract

Tyrosinase is the rate-limiting enzyme critical for melanin synthesis and controls pigmentation in the skin. The inhibition of tyrosinase is currently the most common approach for the development of skin-whitening cosmetics. Gagunin D (GD), a highly oxygenated diterpenoid isolated from the marine sponge *Phorbas* sp., has exhibited cytotoxicity toward human leukemia cells. However, the effect of GD on normal cells and the molecular mechanisms remain to be elucidated. In the present study, we identified for the first time the anti-melanogenic activity of GD and its precise underlying mechanisms in mouse melan-a cells. GD significantly inhibited melanin synthesis in the melan-a cells and a reconstructed human skin model. Further analysis revealed that GD suppressed the expression of tyrosinase and increased the rate of tyrosinase degradation. GD also inhibited tyrosinase enzymatic activity. In addition, GD effectively suppressed the expression of proteins associated with melanosome transfer. These findings suggest that GD is a potential candidate for cosmetic formulations due to its multi-functional properties.

## 1. Introduction

Melanin plays an important role in protecting against the harmful effect of ultraviolet radiation (UVR) and various environmental oxidative stresses [1]. Melanin is synthesized by melanocytes found in the basal layer of the epidermis [2]. In melanosomes, specialized intracellular organelles of melanocytes, melanin is produced by sequential enzymatic processes and is then moved along the melanocyte dendrites and transferred to adjacent keratinocytes for photo-protection [3,4]. Skin color is determined by the production of melanin and the distribution of melanosomes transferred by each melanocyte to its neighboring keratinocytes [3].

Tyrosinase and tyrosinase-related protein 1 (TRP-1) and TRP-2 play an important role in melanin synthesis. Tyrosinase is a type 1 membrane glycoprotein that is the rate-limiting enzyme in melanin biosynthesis as it initially hydroxylates tyrosine to 3,4-dihydroxylphenylalanine (DOPA) and then oxidases DOPA to dopaquinone [3,5,6]. TRP-1 increases the eumelanin/pheomelanin ratio, facilitating the formation of carboxyl group-containing DHICA oxidase eumelanins [7]. TRP-2 acts as a dopachrome tautomerase and catalyzes a quicker conversion of dopachrome to 5,6-dihydroxyindol-2-carboxylic acid (DHICA) [8]. Tyrosinase is degraded endogenously by proteasomes [9]. Ubiquitin-mediated proteasomal degradation plays an important role in the removal of normal protein, as well as in the elimination of misfolded proteins for homeostasis [10]. Therefore, a balance between tyrosinase expression and degradation is considered a new target for skin whitening.

Melanogenesis is involved in various signal transduction pathways. One of the pathways is associated with microphthalmia-associated transcription factor (MITF), which has been demonstrated to upregulate tyrosinase, TRP-1, and TRP-2 through binding to an M box in their promotor sites [11]. The expression of MITF is regulated by many upstream transcription regulators, including paired box 3 (PAX3) and SRY-related HMG-box 10 (SOX10) [12]. Moreover, MITF is also involved in melanosome transfer by regulating the expression of rab27a, a small GTPase of the melanosome transfer complex [13]. The rab27a-melanophilin-myosin Va tripartite complex is essential for the distribution of melanin to basal and suprabasal keratinocytes because the complex aids in the association of melanosomes with actins in melanocyte dendrites [14,15]. The motor protein myosin Va attaches to melanosomes through an interaction with melanophilin and rab27a [15].

Marine natural products including sponges have produced various biologically active and structurally unique secondary metabolites [16]. Gagunin D (GD), one of 17 gagunins, was isolated from the sponge *Phorbas* sp. collected from Gagu-Do, Korea, by Shin’s group. GD also shows a unique carbon skeleton with a highly oxygenated functionality on an unusual 10,13-bis-*epi*-homoverrucosane [17,18]. Although the unique chemical structure is a quite interesting, biological activities remain to be evaluated.

In our continuous efforts to identify bioactive natural products, Gagunin D (GD) from a Korean sponge was identified as a potential modulator of melanogenesis from a screening of marine natural product-derived compounds.

GD, a highly oxygenated diterpenoid, exhibits anti-melanogenic activity through the modulation of tyrosinase expression and degradation and the regulation of melanosome transfer.

## 2. Results

### 2.1. Inhibitory Effect of GD on Melanin Biosynthesis in Melan-a Cells

**Figure 1:**
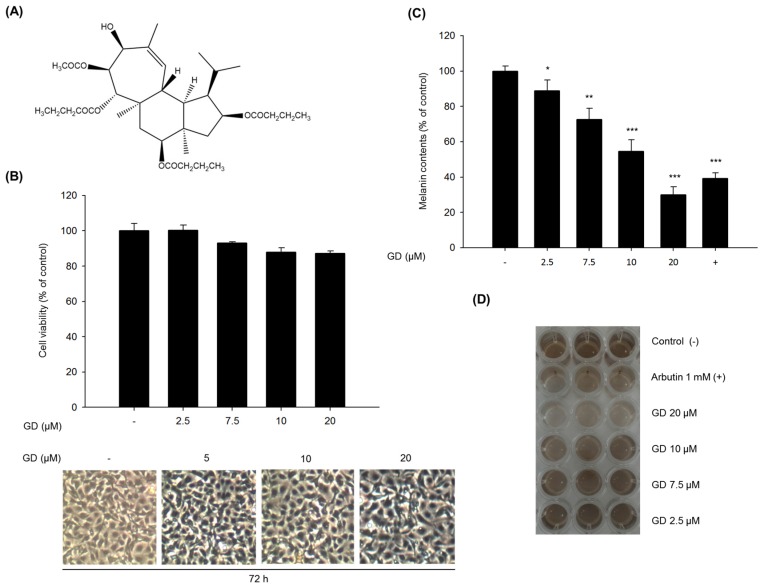
Effects of GD on melanogenesis in melan-a cells. (**A**) The chemical structure of GD; (**B**) Cell viability was determined by MTT assay with the indicated concentrations of GD for 72 h, and the cellular morphology was observed under a phase-contrast microscope (at 100× magnification); (**C**) Inhibition of melanin biosynthesis in melan-a cells treated with the indicated concentrations of GD for 72 h. Arbutin (1 mM) was used as a positive control; (**D**) Macroscopic views of the results of (**C**). Data are shown as the mean ± standard deviation. * P < 0.05, ** P < 0.01, *** *P* < 0.001 are considered statistically significant compared to the control group.

Cell viability was evaluated by MTT assay to determine the cytotoxicity of GD (Figure 1A) on murine melanocytes. Melan-a cells were treated with different concentrations of GD for 72 h. GD did not exhibit significant cytotoxicity (cell viability was 89% at 20 μM GD, Figure 1B), and no remarkable morphological changes were observed. We then investigated the effect of GD on melanin production. GD significantly decreased the melanin content in a concentration-dependent manner, with an IC_50_ of 12.7 μM (Figure 1C). As shown in Figure 1D, the inhibitory activity of 20 μM GD was stronger than that of 1 mM arbutin, a well-known skin-whitening agent.

### 2.2. Effect of GD on Melanogenesis-Related Proteins and Gene Expression

**Figure 2:**
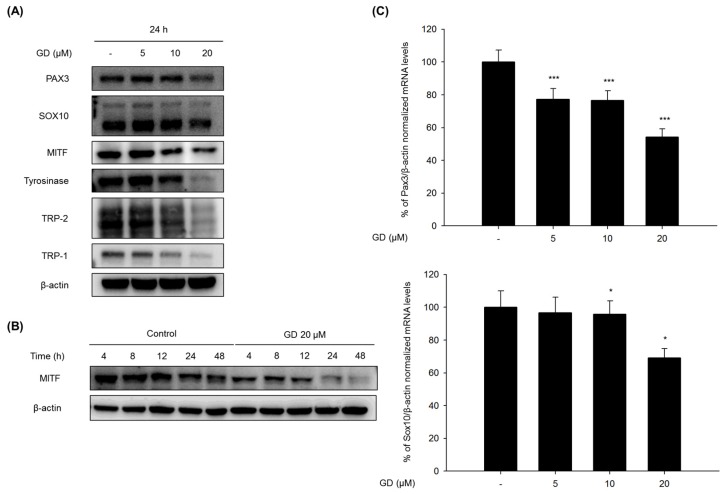

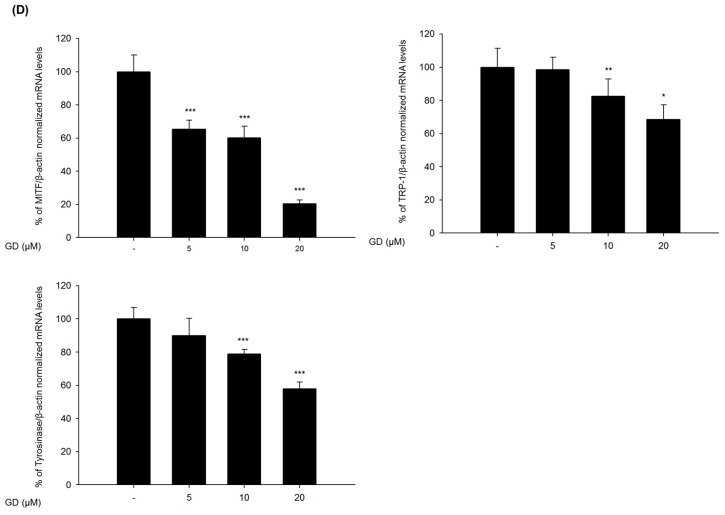
(**A**) Melan-a cells were treated with the indicated concentrations of GD for 24 h, and (**B**) melan-a cells were treated for the indicated times in the presence or absence of 20 μM GD; (**C**,**D**) Melan-a cells were treated at various concentrations of GD for 12 h, and the mRNA levels were examined by real-time RT-PCR. * P < 0.05, ** P < 0.01, *** *P* < 0.001 are considered statistically significant compared to the control group.

Cells were treated with GD for 24 h, and the effects of GD on the expression of melanogenesis-related proteins were analyzed by Western blot to elucidate the mechanisms of action in the inhibition of melanogenesis by GD. GD effectively downregulated the protein levels of PAX3, SOX10, MITF, tyrosinase, TRP-1 and TRP-2 (Figure 2A). The expression of MITF was significantly decreased at 20 μM GD. Additional analysis was performed at various time points after treatment with 20 μM GD. Starting at 4 h, the expression of MITF was significantly downregulated (Figure 2B).

The expression levels of genes associated with the transcription factors of MITF were also evaluated by real-time RT-PCR. GD effectively downregulated the mRNA expressions of PAX3 and SOX10 at 12 h in a concentration-dependent manner (Figure 2C). GD also suppressed the mRNA expression of MITF, tyrosinase and TRP-1 in a concentration-dependent manner (Figure 2D). To confirm the effects of GD on the expressions of the target genes, the dual luciferase assay was performed. GD also effectively suppressed the transcriptional activity of MITF and tyrosinase (Figure S1).

### 2.3. Effect of GD on Tyrosinase Enzyme Activity and Degradation

**Figure 3:**
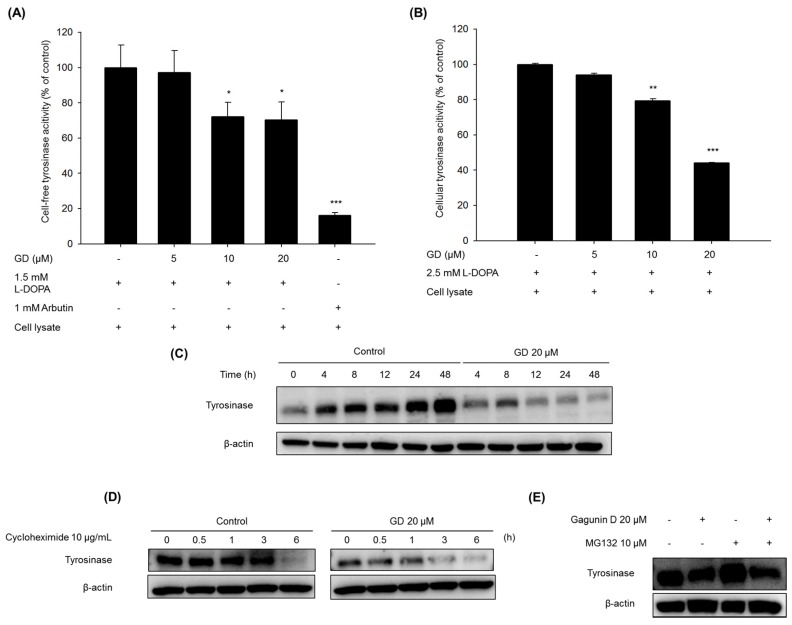
Effects of GD on (**A**) cell-free tyrosinase activity and (**B**) cellular tyrosinase activity. Tyrosinase activity was measured by dopachrome formation from l-DOPA as a substrate. (**C**,**D**,**E**) Effects of GD on the degradation of tyrosinase. (**C**) Melan-a cells were treated for the indicated times in the presence or absence of GD; (**D**) Cells were treated with 10 μg/mL cycloheximide with or without 20 μM GD for the indicated times; (**E**) Cells were treated with or without 20 μM GD for 18 h after pretreatment with 10 μM MG132 for 1 h. * P < 0.05, ** P < 0.01, *** *P* < 0.001 are considered statistically significant compared to the control group.

The effect of GD on tyrosinase enzymatic activity was determined using cell-free lysates from melan-a cells, as described in Materials and Methods. As shown in Figure 3A, GD significantly inhibited the tyrosinase activity at concentrations greater than 10 μM. In addition, cellular tyrosinase activity was also inhibited by treatment with GD (Figure 3B).

When the tyrosinase protein levels were monitored for up to 48 h, GD effectively downregulated the expression of tyrosinase protein in a time-dependent manner compared to the control cells (Figure 3C). To further analyze whether the effect of GD on the expression of tyrosinase proteins was associated with the proteolytic degradation of the proteins, the levels of tyrosinase expression were monitored for up to 6 h after pretreatment with cycloheximide, a protein synthesis inhibitor [19]. Treatment with GD accelerated the proteolytic degradation of tyrosinase compared to the control groups. Next, to examine the degradation pathway by GD, a 26s proteasome inhibitor MG132 was used for pretreatment, and the tyrosinase level was determined by Western blot. As shown in Figure 3E, tyrosinase degradation was abrogated by the proteasome inhibitor, a multicatalytic proteinase complex that selectively degrades intracellular ubiquitinated proteins [6,20]. Thus, GD accelerated the degradation of tyrosinase and inhibited tyrosinase enzymatic activity.

### 2.4. Effect of GD on Melanosome Transfer-Related Proteins

**Figure 4:**
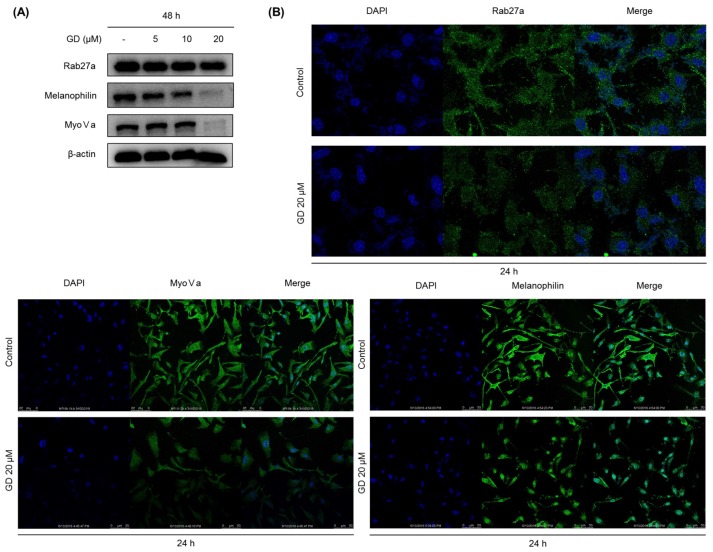
(**A**) Melan-a cells were treated with the indicated concentration of GD for 48 h and melanosome transfer-related proteins were analyzed by Western blot; (**B**) Effect of GD on the protein levels determined by immunocytochemistry.

To further examine the effects of GD on melanosome transfer, the expression of melanosome transfer-associated proteins was determined by Western blot. As depicted in Figure 4A, GD effectively suppressed the expression of rab27a, melanophilin, and myosin Va, which are the main melanosome transfer-associated proteins, after treatment of GD for 48 h (Figure 4A). The downregulation of rab27a, melanophilin and myosin Va by GD was confirmed by immunocytochemical analysis (Figure 4B).

### 2.5. Effect of GD on Melanin Synthesis in a Reconstructed Human Skin Model

**Figure 5:**
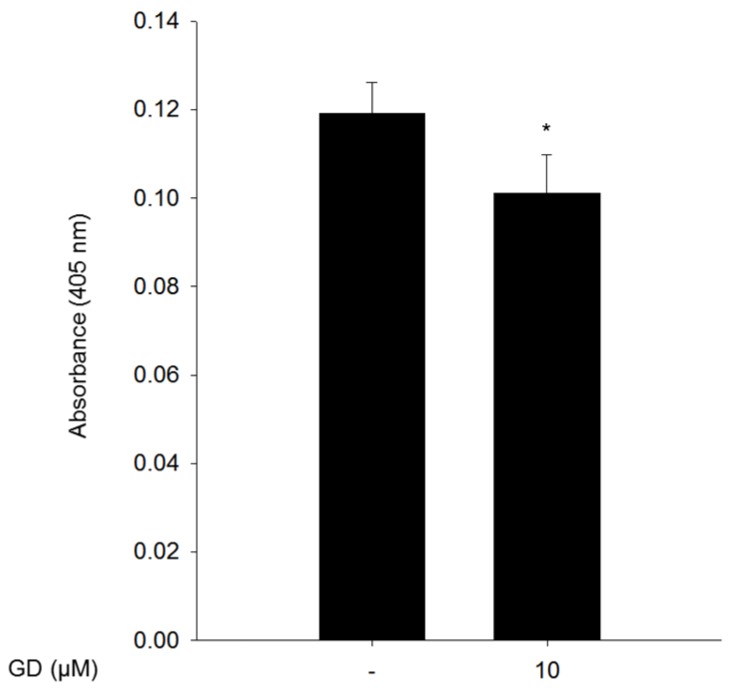
The inhibitory effect of GD on melanin biosynthesis in a reconstructed human skin model, Neoderm^®^-ME. The melanin content from the lysates was measured at 405 nm after dissolving the reconstructed human skins in 1 N NaOH. * P < 0.05 is considered statistically significant compared to the control group.

To further investigate the effect of GD on melanin biosynthesis in human skin, a reconstructed human skin model, Neoderm^®^-ME, was employed, and GD (10 μM) was applied for 72 h before spectrophotometric evaluation. The reconstructed human skin was irradiated with 50 mJ/cm^2^ UVB every three days for a total of two times to induce melanin production [21]. The irradiated human skin was treated with GD for 72 h then the reconstructed human skin was dissolved in 1 N NaOH. The melanin contents were detected by absorbance at 405 nm. GD significantly inhibited melanin biosynthesis in the UVB-irradiated reconstructed human skin model (Figure 5).

## 3. Discussion

Natural product-derived compounds are commonly used in cosmetic formulations [22]. In our continuous efforts to identify bioactive natural products, this study evaluated the anti-melanogenic activity of natural products, and Gagunin D, a marine sponge-derived diterpenoid, was found to be active in the inhibition of melanin synthesis [17,18]. Melanin biosynthesis is a multistep process, including melanin formation and melanosome transfer [23]. Therefore, there are many targets in the evaluation of skin-whitening activity. Inhibition of tyrosinase, one of the main enzymes in melanin biosynthesis, and modulation of MITF, a transcriptional factor, are common approaches in the search for bioactive sources for anti-melanogenic activity [22]. Several natural product-derived compounds, such as arbutin, hydroquinone, niacinamide, have also exhibited anti-melanogenic activity, with targets of tyrosinase and MITF [24]. In addition, regulation of the expression and degradation of tyrosinase and inhibition of melanosome transfer are plausible targets for skin-whitening agents [24,25,26].

Although sponges produce many chemical compounds with widely varying carbon skeletons, the molecular mode of action of most compounds remains unclear [27]. Gagunin A–G, highly oxygenated diterpenoids isolated from the marine sponge *Phorbas* sp., were identified by Shin and have exhibited cytotoxicity toward human leukemia cells [17]. We found that among Gagunin A–G, Gagunin D has the most potent anti-melanogenic effect. However, there has been no previous study on its effect on melanogenesis in melanocytes. Thus, we characterized the anti-melanogenic effect of GD, which does not have the typical structure of conventional hypo-pigmenting agents.

In the present study, we found that GD effectively inhibited melanin biosynthesis in both cultured melanocytes and in a reconstructed human skin model.

The mechanisms of action in the inhibitory activity of melanin synthesis by GD were also elucidated through analysis of melanogenesis-associated biomarkers. GD significantly suppressed the expression of the transcription factor MITF, which led to the suppression of target proteins tyrosinase, TRP-1 and TRP-2, major enzymes of melanin synthesis in melanocytes. Additionally, the downregulation of MITF was associated with the suppressive expression of transcription factors PAX3 and SOX10, which are well-known transcription factors for MITF. The downregulation of MITF and tyrosinase by GD was confirmed by the inhibitory promoter activities of MITF and tyrosinase through a dual luciferase assay. These findings suggest that GD affects both the expression and activity of MITF in the melanin biosynthetic pathway. In terms of targeting tyrosinase by GD, GD effectively suppresses the expression of tyrosinase at the mRNA and protein levels and directly inhibits the enzymatic activity both in cell-free and cell-based systems. These dual functions of GD contribute to the anti-melanogenic activity of GD. We further elucidated the mechanisms associated with the regulation of tyrosinase expression by GD in melanocytes. The downregulation of tyrosinase was correlated with the acceleration of the ubiquitin-dependent proteasomal degradation of tyrosinase. These findings indicate that GD can suppress the level of tyrosinase in cells through the modulation of its expression and the enhancement of degradation.

To obtain successful skin-whitening activity, not only the regulation of de novo melanogenesis but also the modulation of melanin trafficking is an important approach. In general, matured melanin in melanocytes moves into keratinocytes in the skin, in a process called melanosome transfer. To determine whether GD affects melanosome transfer, the biomarkers associated with melanosome transfer were investigated after treatment with GD for 48 h. The protein expression of melanosome-associated biomarkers rab27a, melanophilin and myosin Va was downregulated by GD treatment. These results were confirmed by the immunocytochemical analysis of the corresponding proteins in the cells after treatment with GD. Morphologically, the dendrite-type melanocytes were altered by GD treatment, indicating an effect on characteristic phenomena of the movement of melanocytes.

Taken together, we herein report that Gagunin D, a marine natural product, exhibits anti-melanogenic activity in melanocytes through the potential inhibition of melanogenesis and melanosome transfer activity.

## 4. Materials and Methods

### 4.1. Materials

The test compound Gagunin D (GD, *MW* = 606, Figure 1A) was isolated from a marine sponge, *Phorbas* sp. collected from Gagu-Do, Korea by Shin [17,18]. GD was dissolved in 100% DMSO and stored at −20 °C for subsequent experiments. Dimethyl sulfoxide (DMSO), thiazolyl blue tetrazolium bromide (MTT), bicinchoninic acid (BCA) and copper sulfate were purchased from Sigma-Aldrich (St. Louis, MO, USA). Roswell Park Memorial Institute (RPMI) 1640 medium, fetal bovine serum (FBS), trypsin-EDTA solution (1×), and antibiotic-antimycotic solution (100×) were purchased from Invitrogen (Carlsbad, CA, USA). Gout polyclonal anti-MITF, anti-tyrosinase, anti-TRP-1 and anti-TRP-2 were purchased from Santa Cruz Biotechnology (Santa Cruz, CA, USA). Rabbit monoclonal anti-myosin Va was from Cell Signaling Biotechnology (Danvers, MA, USA). Goat polyclonal anti-melanophilin and mouse monoclonal anti-rab27a were from Abcam (Cambridge, MA, USA).

### 4.2. Cell Culture

Melan-a cells (originally established by Dorothy C. Bennett at the University of London) were provided by the Skin Research Institute, Amore Pacific Co., Seoul, Korea. Melan-a cells were cultured in RPMI 1640 medium supplemented with 10% FBS, 100 units/mL penicillin, 100 μg/mL streptomycin, 250 ng/mL amphotericin B and 20 nM tetradecanoyl phorbol acetate (TPA) at 37 °C in a humidified incubator under an atmosphere containing 10% CO_2_.

### 4.3. Cell Viability Assay (MTT Assay)

MTT solution (final concentration of 500 μg/mL) was added to each well before incubation for 3 h at 37 °C. The medium was removed, dimethyl sulfoxide (DMSO) was added to each well and the absorbance of the dissolved formazan was measured at 570 nm.

### 4.4. Determination of the Melanin Contents

Melan-a cells were plated in 6-well plates at a density of 1.0 × 10^5^ cells/well. After an additional 72 h incubation, cells were treated with various concentrations of the GD for 72 h. After treatment, the cells were washed with DPBS and were dissolved in 1 N NaOH containing 10% DMSO for 1 h at 60 °C. The optical density was measured at 475 nm.

### 4.5. Cell-Free Tyrosinase Activity Assay

A crude enzyme source of solubilized tyrosinase was prepared [28]. Melan-a cells were cultured in 100 mm dishes, and the cells were washed with ice-cold PBS and lysed with 1% (*v*/*v*) Triton-X/phosphate-buffered saline (pH 6.8). Cells were disrupted by freezing and thawing and the lysates were clarified by centrifugation at 12,000 rpm for 5 min at 4 °C. The protein concentration was determined by the Bradford method. To determine the inhibitory effect of the in vitro tyrosinase activity, the reaction mixtures contained 0.1 M phosphate buffer (pH 6.8), 1.5 mM of l-DOPA, the tested concentration of GD, and 40 μg of protein in a total volume of 1 mL. After incubation at 37 °C overnight, dopachrome formation was measured at 475 nm.

### 4.6. Cellular Tyrosinase Activity Assay

l-DOPA was used as a substrate, and the tyrosinase activity was examined by measuring dopachrome formation at 475 nm. Melan-a cells were cultured in 100 mm dishes and treated with various concentrations of GD for 24 h. The cells were washed with ice-cold PBS and lysed with 1% (*v*/*v*) Triton-X/phosphate-buffered saline (pH 6.8). Cells were disrupted by freezing and thawing and the lysates were clarified by centrifugation at 12,000 rpm for 5 min at 4 °C. The protein concentration was determined by the Bradford method. The reaction mixture was composed of 40 μg protein (adjusted to 100 μL with 0.1 M PBS, pH 6.8) and 100 μL of 5 mM l-DOPA was added to each well of a 96-well plate. After incubation at 37 °C for 15 min, the absorbance was measured at 475 nm. The percentage of tyrosinase inhibition was calculated as [1 − (sample OD/control OD)] × 100.

### 4.7. Analysis of Protein Expression by Western Blot

Melan-a cells were treated with different concentrations of GD for the indicated times. The cells were lysed with extraction buffer, and the protein concentrations were determined using the BCA method. The proteins from the cell lysates were loaded onto 10% SDS-polyacrylamide gels and then transferred to PVDF membranes (Millipore, Bedford, MA, USA). Nonspecific binding was blocked with 5% BSA in TBS containing 0.01% Tween-20 for 1 h at room temperature before incubation with diluted primary antibodies (1:1000 or 1:500) overnight at 4 °C. Then, the membranes were incubated with specific secondary antibodies (1:1000) for 1 h and washed three times with TBS-Tween 20. The expression of β-actin was used as an internal standard. Membranes were subjected to WestZol (iNtRON Biotechnology, Gyeonggi-do, Korea) and were visualized using the LAS-4000 imaging system (Fuji film Corp., Tokyo, Japan).

### 4.8. Analysis of Gene Expression by Real-Time RT-PCR

Melan-a cells were cultured in 100 mm dishes and treated with GD for 12 h. Total RNA was isolated using TRIzol Reagent (Invitrogen, Grand Island, NY, USA) and was reverse-transcribed using a Reverse Transcription System (Promega, MI, USA) according to the manufacturer’s instructions. Specific gene primers were designed and synthesized by Bioneer Corporation (Daejeon, Korea): MITF forward, 5′-CTAAGTGGTCTGCGGTGTCTC-3′; MITF reverse, 5′-GGTTTTCCAGGTGGGTCTG-3′; TYROSINASE forward, 5′-CACCCTGAAAATCCTAACTTACTCA-3′; TYROSINASE reverse, 5′-CTCTTCTGATCTGCTACAAATGATCT-3′; TRP-1 forward, 5′-TGGGAACACTTTGTAACAGCA-3′; TRP-1 reverse, 5′-ACTGCTGGTCTCCCTACATTTC-3′; SOX10 forward, 5′-GAAGCCCCACATCGACTTCG-3′; SOX10 reverse, 5′-GGCAGGTATTGGTCCAGCTC-3′; PAX3 forward, 5′-CCGGGGCAGAATTACCCAC-3′; PAX3 reverse, 5′-GCCGTTGATAAATACTCCTCCG-3′; β-actin forward, 5′-AAGGCCAACCGTGAAAAGAT-3′; β-actin reverse, 5′-GTGGTACGACCAGAGGCATAC-3′; Real-time PCR was conducted using a CFX Connect Real-Time PCR Detection System (Bio-Rad, Hercules, CA, USA). Each PCR amplification included 5 μL of reverse transcription product, iQ SYBR Green Supermix (Bio-Rad, Hercules, CA, USA), and primers in a total volume of 20 μL. The following conditions were used: 95 °C for 20 s prior to the first cycle; 40 cycles of 95 °C for 20 s, 56 °C for 20 s, and 72 °C for 30 s; 95 °C for 1 min; and 55 °C for 1 min. All experiments were performed in quadruplicate, and the analysis was performed using the comparative C_T_ method with β-actin used for normalization.

### 4.9. Immunocytochemistry

Melan-a cells were grown on coverglass-bottom dishes coated with 0.2% gelatin. After treatment with GD, the cells were fixed with 4% paraformaldehyde in PBS for 15 min. After blocking with 1% BSA in PBS for 30 min at room temperature, the cells were incubated with primary antibody at 4 °C overnight. Following incubation, the cells were incubated with fluorescence-conjugated secondary antibody for 2 h at room temperature. DAPI (0.5 mg/mL) was used to counterstain the nuclei. Images were acquired using a Leica confocal micro system (Wetzlar, Germany).

### 4.10. Transient Transfection and Dual Luciferase Assay

pMITF-Gluc and pTYR-Gluc reporter systems harboring the promoter region of MITF and TYR were provided by Amore Pacific R&D Center (Seoul, Korea). pGL3-FL was obtained from S. Oh (Inje University, Busan, Korea). At 40%–50% confluency, the cells were washed with DPBS, and the Gaussia luciferase reporter construct pMITF-Gluc, pTYR-Gluc, and the control firefly luciferase vector (pGL3-FL) were transfected using FuGENE^®^ HD Transfection Reagent (Promega, Madison, WI, USA). After a 24 h incubation with GD, the cell lysates were prepared and the Gaussia and firefly luciferase activity were determined by the Gaussia luciferase assay kit (New England BioLabs, Ipswich, MA, USA) and luciferase reporter assay system (Promega, Madison, WI, USA), respectively, according to the manufacturers’ protocols, using a luminometer (MicroLumat Plus, Berthold Technologies, Dortmund, Germany).

### 4.11. Reconstructed Human Skin Model

The reconstructed human skin model, Neoderm^®^-ME, was purchased from Tego Science (Seoul, Korea) and was analyzed according to the manufacturer’s instructions. The reconstructed human skin was irradiated with 50 mJ/cm^2^ UVB every three days using BIO-SUN (Vilber Lourmat, Marne, France) for a total of two times to induce melanin production. After treatment of 10 μM GD for 72 h, the Neoderm^®^-ME was dissolved in 1 N NaOH and sonicated, and the absorbance of the supernatants at 405 nm was measured.

### 4.12. Statistical Analysis

The data are presented as the mean ± standard deviation (SD) for independently performed experiments. The statistical significance (*P* < 0.05) was analyzed using Student’s *t*-test. One-way analysis of variance (ANOVA) was used to compare the mean responses among treatments.

## 5. Conclusions

In this study, Gagunin D, a highly oxygenated diterpenoid, was identified as an inhibitor of melanin biosynthesis and melanosome transfer in melanocytes. GD also downregulated the expression of transcription factors associated with the regulation of tyrosinase and its target proteins without affecting the cytotoxicity in melanocytes. These findings suggest that the anti-melanogenic activity of GD may be considered to serve as a plausible candidate for a skin-whitening agent.

## Supplementary Materials

The following is available online at www.mdpi.com/1660-3397/14/11/212/s1, Figure S1: The effects of GD on the transactivation of MITF and tyrosinase in melan-a cells measured by transient transfection and a dual luciferase assay.
