# Supplementary material for: Anti-Melanogenic Activity of Gagunin D, a Highly Oxygenated Diterpenoid from the Marine Sponge Phorbas sp., via Modulating Tyrosinase Expression and Degradation
Source: Mar Drugs. 2016 Nov 17;14(11):212. doi: 10.3390/md14110212 (PMC5128755; doi:10.3390/md14110212)
Supplement: Supplementary file 1 [file marinedrugs-14-00212-s001.docx]

Supplementary Materials: Anti-Melanogenic Activity of Gagunin D, a Highly Oxygenated Diterpenoid from the Marine Sponge *Phorbas* sp., via Modulating Tyrosinase Expression and Degradation

**Ho Yeon Lee, Eun Jeong Jang, Song Yi Bae, Ju-eun Jeon, Hyen Joo Park, Jongheon Shin and
Sang Kook Lee**


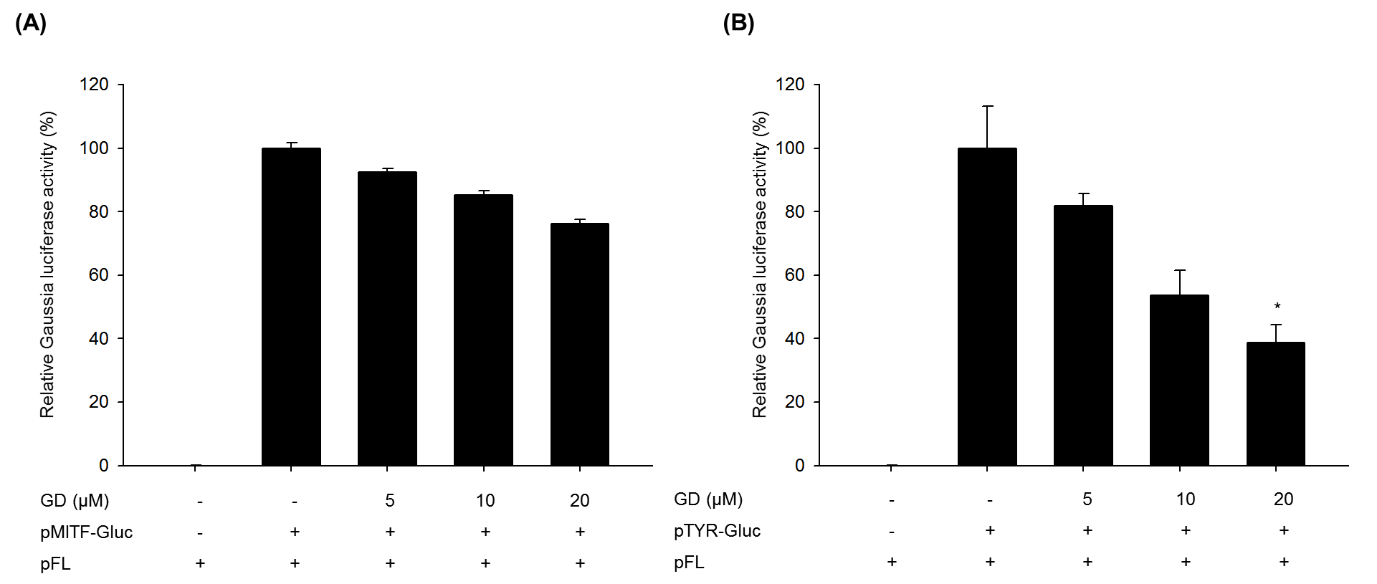


**Figure S1.** The effects of GD on the transactivation of MITF and tyrosinase in melan-a cells measured by transient transfection and a dual luciferase assay. Melan-a cells were transfected with Gaussia luciferase reporter constructs, pMITF-Gluc (**A**) and pTyrosinase-Gluc (**B**) plasmid and treated with the indicated concentrations of GD for 24 h. The cells were co-transfected with the firefly luciferase control vector (pFL) to normalize the transfection rates. * *P* < 0.05, are considered statistically significant compared to the control group.
